# Immune surveillance of cytomegalovirus in tissues

**DOI:** 10.1038/s41423-024-01186-2

**Published:** 2024-08-12

**Authors:** Andrea Mihalić, Jelena Železnjak, Berislav Lisnić, Stipan Jonjić, Vanda Juranić Lisnić, Ilija Brizić

**Affiliations:** 1 Center for Proteomics, Faculty of Medicine, University of Rijeka, Rijeka, Croatia; 2 Department of Biomedical Sciences, Croatian Academy of Sciences and Arts, Rijeka, Croatia

**Keywords:** Cytomegalovirus, Immune response, Pathogenesis, Tissue, MCMV, HCMV, Viral infection, Antimicrobial responses, Infection

## Abstract

Cytomegalovirus (CMV), a representative member of the *Betaherpesvirinae* subfamily of herpesviruses, is common in the human population, but immunocompetent individuals are generally asymptomatic when infected with this virus. However, in immunocompromised individuals and immunologically immature fetuses and newborns, CMV can cause a wide range of often long-lasting morbidities and even death. CMV is not only widespread throughout the population but it is also widespread in its hosts, infecting and establishing latency in nearly all tissues and organs. Thus, understanding the pathogenesis of and immune responses to this virus is a prerequisite for developing effective prevention and treatment strategies. Multiple arms of the immune system are engaged to contain the infection, and general concepts of immune control of CMV are now reasonably well understood. Nonetheless, in recent years, tissue-specific immune responses have emerged as an essential factor for resolving CMV infection. As tissues differ in biology and function, so do immune responses to CMV and pathological processes during infection. This review discusses state-of-the-art knowledge of the immune response to CMV infection in tissues, with particular emphasis on several well-studied and most commonly affected organs.

## Introduction

Human cytomegalovirus (HCMV) is a widely prevalent beta-herpesvirus that infects between 40% and >90% of individuals in a given population, with higher seroprevalence noted among women, older individuals, and individuals with a lower socioeconomic status [1]. In immunocompetent individuals, primary HCMV infection and subsequent reactivation typically produce no symptoms and are effectively controlled by the immune system. However, in immunologically immature newborns or fetuses, HCMV is a major viral causative agent of congenital infections, which can result in stillbirth or cause numerous neurological sequelae [2]. HCMV is also a significant opportunistic pathogen in immunocompromised individuals, such as organ transplant recipients and patients with AIDS [3]. Finally, infection with HCMV has been associated with numerous other diseases, including atherosclerosis and immunosenescence, as well as with premature mortality [4].

Host-to-host transmission of HCMV occurs primarily through saliva, either by direct contact or inhalation of droplets. The virus can also be transmitted sexually [5], through blood or organ transplantation [3], or transplacentally from the mother to a fetus [2]. In addition, newborns can be perinatally infected by exposure to secretions in the birth canal or via breast milk contaminated with HCMV [6]. Young children secrete HCMV in saliva and urine for prolonged periods of up to several years [7]. During primary HCMV infection, the virus spreads via peripheral blood to various organs [8, 9]. Efficient HCMV dissemination and infection of different cell types and tissues are facilitated by broad cellular tropism, enabled by interactions between viral entry gH/gL glycoprotein complexes and surface receptors on host cells [10]. The entry receptors for HCMV are platelet-derived growth factor receptor-α (PDGFRα), which mediates the infection of fibroblasts, and neuropilin-2 (Nrp2), which is essential for endothelial and epithelial cell infection [11–13].

Unsurprisingly, a wide spectrum of clinical symptoms can be observed in individuals with HCMV disease, with hepatitis, neurologic sequelae, retinitis, enterocolitis, and pneumonitis being the most frequent [14]. Like all herpesviruses, HCMV establishes lifelong latency, a nonproductive phase of infection with restricted expression of viral genes punctuated with occasional reactivations [15]. Latent HCMV infection appears to be established primarily in CD34^+^ hematopoietic progenitor cells (HPCs), and reactivation of the latent virus is triggered by cellular differentiation and inflammation [16]. Consequently, HCMV is especially perilous for immunocompromised individuals. Indeed, viral reactivation is a common complication and risk factor following organ transplantation [3].

An orchestrated response of both innate and adaptive immunity is required to control the infection caused by cytomegaloviruses (CMVs) [17]. Multiple layers of innate immune mechanisms restrict CMV infection. Specifically, type I interferons (IFNs) and NK cells play crucial roles during early infection [18, 19], and CD8 and CD4 T cells play crucial roles during the late phase of acute infection. Cytotoxic CD8 T cells are required to restrict the viral replication in most tissues [20]. In contrast, CD4 T cells resolve persistent infection at sites important for horizontal virus spread, most notably in the salivary glands [21]. More than half of the >170 HCMV genes are involved in immune evasion. Like other CMVs, HCMV efficiently modulates numerous immune mechanisms owing to its millennia of coevolution with its hosts [17, 22]. Consequently, the virus persists in the organism, but the immune system represses its active replication. Constant immunosurveillance requires extensive resources, as exemplified by the finding that more than 10% of T cells present in the blood of HCMV-seropositive individuals are often specific for HCMV [20]. Tissue-resident HCMV-specific T cells are also found at significant frequencies in various tissues [23].

The importance of tissue-specific immune responses and pathogenesis is quickly becoming an important field of research, and this information is required both for understanding the fundamental principles of virus pathogenesis and control and is a mandatory prerequisite for developing better treatments, disease management, and vaccine strategies. As tissues differ in biology and function, so do the immune responses to CMV. HCMV exhibits strict species specificity and, for that reason, cannot be used in investigations of the immune responses and pathogenesis of infection in laboratory animals. Furthermore, tissue-specific immune responses to HCMV are challenging to assess in humans, which, together with species-specific tropism, necessitates the use of animal CMVs and their natural hosts. Of those, the most common and well-established are the experimental systems that employ the infection of mice with genetically and biologically related mouse CMV (MCMV) to model HCMV disease (reviewed in [24–26]). Therefore, the primary focus of this review, along with tissue-specific immune responses to HCMV, will be tissue-specific immune responses following MCMV infection in mice.

### Mouse model of CMV infection

MCMV has been successfully used as a model for HCMV disease for decades given its numerous similarities [27]. Herpesvirus infection in mammals preceded divergence between primates and rodents; thus, basic concepts of virus‒host interactions have been largely preserved. Differences and loss of sequence conservation likely result from coevolution with respective hosts [28]. The MCMV and HCMV genomes are largely collinear, with many homologous genes. Multiple strains of both MCMV and HCMV exist in nature, and laboratory isolates acquire mutations during in vitro propagation [24–26]. Both viruses have broad cell tropism and establish latent infection, and numerous aspects of HCMV pathogenesis have been successfully modeled in mice infected with MCMV. Notably, there are substantial overlaps in the immune response to HCMV and MCMV infection in humans and mice, respectively.

However, differences between HCMV and MCMV also need to be considered. The organization of their genomes differs in several aspects, and both viruses possess a unique set of genes. The broad tropism of HCMV is secured by two gH/gL complexes, namely, gH/gL/gO and gH/gL/pUL128-131 (pentameric complex) [29]. MCMV has a homologous gH/gL/gO complex and an alternative complex, gH/gL/MCK-2 [30, 31]. Both MCK-2 and UL128 have CC-chemokine domains, but it is unclear to what extent the functions of these two gH/gL complexes overlap [29]. Notably, both HCMV and MCMV lacking gH/gL complexes cannot produce infectious particles and therefore cannot spread efficiently, indicating the critical role of these proteins in infection. Mouse studies have suggested that the complex with gO is required for infection of the first target cells, whereas both gH/gL complexes mediate intratissue spread following the infection of initial cells [32]. Similarly, both MCMV and HCMV encode the positional homologs UL116/M116, which interact with gH and likely serve as chaperones [33–35].

Differences exist in the entry receptors used by these viruses: while HCMV uses PDGFRα and Nrp2 as entry receptors [11–13], MCMV requires Nrp1 and MHC-I molecules for entry [36, 37]. In contrast to HCMV, which is considered to establish latency in HPCs, myeloid progenitors, and monocytes, MCMV can establish latency in fibroblasts, endothelial cells, and macrophages in different tissues (reviewed in [15]). However, the lack of evidence for HCMV latency in other cell types does not exclude the ability of HCMV to establish latency in other cells.

Mouse strains can be divided into MCMV-sensitive and MCMV-resistant strains based on the ability of their NK cells to recognize infected cells by activating receptors (reviewed in [38]). Certainly, the most well-known example is C57BL/6 mice, and the activating Ly49H receptors of these mice recognize the virally encoded m157 protein. However, it is important to note that the Ly49H receptor from C57BL/6 mice cannot recognize m157, which is encoded by most tested MCMV strains [39], indicating strong evolutionary selection. Similarly, activated NK cell expansion in humans is dependent on KIR receptors, which are functionally related to Ly49 receptors [40], as well as to the NKG2C receptor recognizing HLA-E molecules (reviewed in [41, 42]).

Finally, in experimental models, MCMV is most commonly administered via systemic, intraperitoneal (i.p.) or intravenous (i.v.) routes, which might not reflect the most common route of HCMV infection, which occurs via mucosal surfaces. Currently, an increasing number of studies are employing the intranasal route of infection along with footpad infection, which is also thought to represent a natural mode of MCMV spread through biting. Importantly, administration of MCMV via different routes results in differences in virus spread and viral loads in tissues [43], which is also likely the case in humans.

## Immune response to Cmv in lymphoid organs

When CMV infects the host, it encounters a complex network of immune cells localized throughout various tissues and organs aimed at controlling the virus. Among them, lymphoid organs are crucial for orchestrating immune defense by acting as specialized sites where immune cells develop, differentiate, and coordinate responses against invading viruses. The spleen and lymph nodes (LNs), secondary lymphoid organs, consist of organized compartments that capture pathogens and support efficient immune responses in a time-effective manner [44]. Therefore, not surprisingly, these organs are among the most studied organs during CMV infection. LNs are critical for preventing viral spread to other organs and are initiators of the immune response [45]. The highly vascularized spleen, which acts as a blood filter and a reservoir for various immune cells, is another target of early CMV infection. MCMV models of i.v. or i.p. infection are typical examples in which viral particles are injected directly into the blood or peritoneal cavity, enabling the virus to quickly reach the spleen via the bloodstream [46, 47]. The presence of the virus induces substantial remodeling of the splenic microarchitecture and initiates a series of coordinated waves of immune cell activation and local and systemic cytokine production, leading to virus control (reviewed in [48]). CMV infection also impacts primary lymphoid organs. In the bone marrow, a primary site for hematopoiesis, CMV infects a wide range of cells and can interfere with hematopoiesis [49–51]. Finally, despite effective immune activation and subsequent control in various lymphoid organs, CMV manages to establish latency in the bone marrow, spleen, and LNs (reviewed in [16, 52]). The focus of this chapter will be directed toward LNs, spleen, and bone marrow, which are crucial for the resolution of productive infection and the formation of long-term immune responses.

### Lymph nodes

LNs are not only key sites of the immune response but can also be infected by CMV. Mature dendritic cells (DCs) are instrumental in the efficient cross-communication between NK and T cells in LNs and in the efficient immune response to CMV. Both HCMV and rhesus monkey CMV (RhCMV) encode a viral homolog of the key anti-inflammatory cytokine IL-10 (reviewed in [53]). Like native IL-10, HCMV IL-10 [54] and RhCMV IL-10 [55] also strongly inhibit DC maturation and reduce IL-12 production by DCs, which is necessary for T-cell priming and NK cell activation. Viral inhibition of DC maturation leads to a long-lasting deficit in adaptive antiviral immunity [54, 55].

NK cells are typically present in low numbers (0.2–0.4%) within the LNs of naive mice or healthy humans. However, upon infection or immunization, more NK cells enter the LN, interact with DCs and T cells, and modulate adaptive immune responses [56–59]. Unlicensed, Ly49-negative (uneducated) NK cells (reviewed in [60]) constitute a majority of the NK cell population in draining LNs shortly after i.p. MCMV infection [61]. These NK cells exhibit increased production of GM-CSF, stimulating DC expansion and activation and ultimately leading to enhanced and maintained antigen-specific T-cell responses. In contrast, licensed (educated) NK cells, which possess inhibitory Ly49 receptors for self-MHC-I, preferentially migrate to infected parenchymal tissues, produce IFNγ, and mediate direct antiviral responses [61]. LN-resident NK cells are also required for the formation of fully functional humoral responses to MCMV [62]. The absence of the activating receptor NCR1 leads to impaired NK cell maturation and function, as well as NK cell migration to regional LNs following i.p. infection, resulting in reduced CD4 + T-cell activation, impaired generation of follicular helper T cells (Tfhs), and inferior maturation of germinal center (GC) B cells. Consequently, fewer antibody-secreting cells were observed in the LNs, and lower amounts of MCMV-specific antibodies were detected in the sera of infected NCR1-deficient mice [62]. Additionally, MCMV limits the production of MCMV-specific antibodies by inducing ICOSL downregulation via the virally encoded m138 protein [63]. ICOSL downregulation on DCs reduces Tfh and GC B-cell numbers in the spleen and LNs. Therefore, the interaction between MCMV and NK cells enhances adaptive immunity by activating DCs and shaping the humoral response, which is partially limited by MCMV evasion mechanisms.

LNs also play a supporting role in CD8 T-cell memory inflation, a process unique to CMV that results in the accumulation of a distinct pool of virus-specific memory CD8 T cells during latency that expand over time (reviewed in [64]). Memory inflation depends on antigen presentation mediated by nonhematopoietic cells in LNs, which serve as a major site of MCMV latency [65, 66]. Inflationary CD8 T cells exhibit a central-memory phenotype (T_CM_) in LNs [65]. Inflationary CD8 T cells with an effector phenotype accumulate in peripheral tissues without demonstrating increased local proliferation. These inflationary effector CD8 T cells are maintained mainly by a population of cells expressing TCF1^+^, the expression of which is regulated by IL-12 and type I IFNs [67]. It has been suggested that a portion of T_CM_ CD8 T cells in LNs might undergo differentiation into effector cells, followed by the migration of newly formed effectors to peripheral tissues, where they control local viral reactivation without further proliferation [65]. However, another study showed that most inflationary effector T cells in the periphery are primarily generated and subsequently maintained by antigen exposure in the blood and latently infected endothelial cells but not in the LNs [68]. Similarly, although HCMV-specific effector T cells predominantly accumulate in the blood rather than in the LNs of humans, research suggests that during reactivation, HCMV-specific CD8 T-cell clones found in peripheral blood rarely originate from the HCMV-specific CD8 T-cell pool in LNs. This result indicates that precursor cells for these peripheral blood CD8 effector-type T cells may arise from other secondary lymphoid tissues or the naive CD8 T-cell pool [69]. However, further studies are needed to confirm this observation. Interestingly, despite hypotheses that the inflationary T-cell response to CMV could alter the subsequent response to other pathogens, previous MCMV infection did not adversely affect cellular or humoral immune responses in mice infected with *Listeria monocytogenes* or West Nile virus [70]. Similarly, previous HCMV infection in humans did not interfere with the total number of memory CD8 T cells specific for EBV or influenza viruses in LNs [71, 72].

Research on other immune cell populations in the LNs of rats revealed the accumulation of γδ T cells in regional popliteal LNs starting two days after the injection of rat CMV (RCMV) into the footpad, which significantly inhibited virus replication and spread. These γδ T cells proliferate in response to IL-2, express high levels of IFNγ, and are able to clear CMV-infected fibroblast monolayers [73]. Moreover, γδ T-cell proliferation was also detected in LNs following i.p. MCMV infection in mice lacking CD8 T and B cells [74].

As shown during MCMV infection, LNs and their associated immune cells act as crucial checkpoints in CMV dissemination to other parts of the host. LNs are crucial for restricting the transition from localized to systemic MCMV infection. Moreover, MCMV utilizes myeloid cells located in LNs as a means of virus dissemination (reviewed in [75, 76]); however, the exact cell type and mode of dissemination depend on the infection route. Following i.p. injection, MCMV first migrates as a cell-free virus from the injection site to the mediastinal LNs, where it infects CD169-positive macrophages of the subcapsular sinus (SSMs). From there, the virus enters the thoracic duct and subsequently the bloodstream, infecting blood monocytes and eventually reaching the spleen, liver, and other organs [46, 77]. MCMV inoculated into the footpad spreads via monocytes [78]. Footpad-inoculated MCMV first reaches the popliteal LNs, where its dissemination depends on infection by CD169^+^ SSMs, which subsequently slow viral spread by shielding permissive fibroblasts [45]. Moreover, viral dissemination via the SSM in the subcapsular sinus is negatively regulated by type I IFNs [79]. Blocking type I IFNs leads to increased SSM infection in LNs, which particularly affects fibroblastic reticular cells (FRCs) and accelerates viral spread to the spleen. Furthermore, inflammatory signals recruit NK cells to the subcapsular sinus as a second line of defense, where they eliminate infected FRCs. Plasmacytoid DCs (pDCs) also contribute to type I IFN production, which is likely triggered by infected cell debris and cytokines from infected FRCs [79]. MCMV administered intranasally enters a new host via olfactory neurons and spreads through infected DCs. A proportion of infected DCs migrate to draining LNs via afferent lymphatic vessels by intravasation to present antigens to T cells. Although it was previously thought that LNs are the last stop for these DCs, it seems that some may reenter the blood circulation through high endothelial venules (HEVs) and, therefore, can disseminate into other tissues, particularly the salivary glands [80]. This process defies normal DC trafficking given that naive DCs usually exit LNs through efferent lymph vessels and not through HEVs. This alternative migration during MCMV infection depends on the viral chemokine receptor M33 and the host adhesion receptor CD44 [80–82]. The HCMV homolog US28 seems to facilitate DC exit from LNs, suggesting that HCMV might utilize the same route [83]. However, more research is needed to understand how HCMV enters the host and which cell types it infects first. This is especially important for vaccine development, which relies on preventing initial infection and boosting the immune response at viral entry and dissemination sites.

### Spleen

The spleen is a major lymphoid organ that functions as an orchestrator of the immune response and often represents the first line of defense against infection. HCMV infection can occasionally trigger splenomegaly and splenic infarctions in adults [84], indicating the significant involvement of this organ in the pathogenesis of CMV infection. In mouse models, the spleen is also a crucial target in the early days post infection (p.i.), and virus spread to distant organs is notably diminished in mice that have undergone splenectomy [85]. In healthy mice and humans, the spleen is divided into the red pulp, which harbors erythrocytes, NK cells, and various myeloid cells, and the white pulp, which encircles the central arteriole and comprises lymphocyte clusters arranged into a T-cell zone and B-cell follicles. Red and white pulp are separated by the marginal zone (MZ), which contains specific subsets of B cells, macrophages, and dendritic cells (reviewed in [86]). MCMV infects the stroma of both red and white pulp and has a particular affinity for endothelial cells [46, 47].

**Fig. 1:**
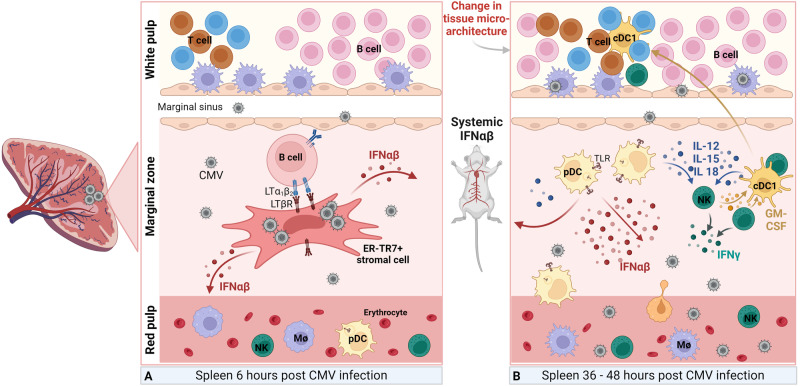
Immune response to CMV infection in spleen. **A** MCMV primarily enters the spleen through the marginal zone around 6 h p.i. where it infects ER-TR7+ stromal cells and activates the LTβR pathway, which depends on the interaction between LTβR-expressing stromal cells and LT-αβ+ B cells. Activation of the LTβR pathway induces NF-κB signaling in infected stromal cells, resulting in the production of type I interferons (IFNαβ). **B** At 36-48 h p.i. MCMV spreads into the red pulp and, in MCMV-sensitive strains, infiltrates the white pulp as well. pDCs from the red pulp start accumulating in the MZ, detecting MCMV via a TLR-dependent mechanism, leading to sustained production of IFNαβ locally and systemically. IFNαβ and IL-12, IL-15, and IL-18, also produced by pDCs and other immune cells, promote NK cell cytotoxicity. At 48 h p.i. there is a widespread infection in the spleen and changes in the spleen’s microarchitecture are starting to be evident. cDC1s form clusters with activated NK cells in an XCR1-dependent manner, delivering IL-12 and IL-15 to NK cells directly. Consequently, NK cells secrete GM-CSF, promoting the re-localization of cDC1 into the T cell zone of the white pulp, where they initiate the priming of CD8 T cells

MCMV induces substantial remodeling of the splenic microarchitecture during the early stages p.i. that is characterized by the loss of macrophages in the marginal zone and the distinct segregation of T- and B-cell compartments [46, 47]. MCMV first infects the marginal zone 6 h after i.p. infection (Fig. 1A), followed by dissemination to the red pulp 17 h p.i. Widespread infection and changes in the splenic microarchitecture become evident at 48 h p.i. and are strongly apparent at 72−96 h p.i. [46, 47] (Fig. 1B). NK cells and type I IFNs are crucial for preventing the destruction of white and red pulp areas in the early days of infection. The absence of NK cells results in the destruction of the T zone stroma with almost complete loss of DCs and T cells [87]. The mechanism involves activation and CXCR3-mediated migration of NK cells from the red to white pulp, where they protect podoplanin-expressing stromal cells by producing perforin and IFNγ, which stimulate the adaptive T-cell response [87, 88]. Furthermore, splenic restructuring involves transient transcriptional repression of the secondary lymphoid chemokine CCL21, which is expressed by splenic stromal cells [47]. The absence of CCL21 expression coincides with the compromised ability of T lymphocytes to localize within the T-cell zone effectively. On the other hand, MCMV infection triggers the activation of the lymphotoxin-β receptor (LTβR) pathway, which partially restores CCL21 expression. CCL21 expression not only helps T lymphocytes position themselves correctly within the white pulp [47] but also controls the initial type I IFN response to MCMV [89].

Type I IFN signaling is a critical constitutive component of innate immunity during early MCMV infection because it can directly inhibit virus replication and activate NK and other immune effector cells [90, 91]. Type I IFN production occurs in three distinct phases in the first 48 h p.i. (Fig. 1) (reviewed in [48]). MCMV predominantly infiltrates the marginal zone of the spleen following i.p. infection, where it infects stromal cells, which subsequently initiate the secretion of type I IFN, marking the onset of the first phase of type I IFN production that is detectable approximately 6−8 h p.i. (Fig. 1A) and diminishes by 24 h. This process relies on signaling between B cells expressing lymphotoxin (LT) αβ and stromal cells expressing LTβR [89]. This initial wave of type I IFN production facilitates NK cell cytotoxicity and NKT cell activation, and these effects are subsequently amplified at later time points [92, 93]. Following the first round of MCMV replication within infected organs after 36 h, pDCs originating from the red pulp begin to accumulate in the MZ (Fig. 1B). These pDCs generate the second, most substantial wave of type I IFN in a TLR-dependent manner [94–98]. The third wave, characterized by a lower intensity than noted in the previous waves, is likely orchestrated by conventional dendritic cells (cDCs) and occurs at approximately 44−48 h p.i. [97, 99]. Similarly, cDC1s secrete IL-12 and IL-15 upon MCMV infection, and their colocalization with activated NK cells in the MZ in an XCR1-dependent manner allows them to deliver these cytokines to NK cells directly. GM-CSF produced by NK cells is delivered to cDC1s, which leads to CCR7-dependent migration of cDC1s into the T-cell zone, where they prime CD8 T cells and boost the adaptive immune response [100]. The extent of the innate and adaptive immune responses against MCMV within the spleen depends on factors such as viral dosage or mouse strain. For example, in C57BL/6 mice, the presence of Ly49H^+^ NK cells enables effective control of the virus in the spleen by day four p.i. This finding contrasts with that in MCMV-sensitive mice such as BALB/c mice, where the viral load remains elevated primarily due to the virus immune evasion mechanisms that inhibit NK cell responses (reviewed in [38, 42, 60]). Together with CD8 T cells and NK cells, CD4 T cells suppress MCMV replication and maintain a state of latency presumably through the local secretion of TNFα and IFNγ given that the depletion of either of these populations results in the reactivation of the virus [101, 102].

Similar to HCMV infection, MCMV infection also results in splenomegaly, which is a consequence of hematopoiesis at fetal hematopoietic sites (extramedullary hematopoiesis [EMH]) in acutely infected adult mice [103, 104]. This phenomenon is dependent on NK cell-mediated cytotoxicity via perforin, interactions between Ly49H and m157, interactions between the NKG2D receptor and its ligands [105], and STAT1 signaling on myeloid cells, and serves to restrict early replication of MCMV in the spleen and promote EMH [106].

### Bone marrow

In vivo and in vitro studies have demonstrated that HCMV can infect bone marrow cells [49, 50]. Clinical HCMV isolates can infect bone marrow precursors in vitro [50], and HCMV DNA can be detected in monocytes differentiated from CD34^+^ precursor cells [107]. Furthermore, CD34^+^ HPCs are latent HCMV sites that can reactivate upon the differentiation of infected monocytes into macrophages in different tissues [10, 16, 50, 107, 108]. HCMV-specific memory T cells have been found in the bone marrow of HCMV-seropositive individuals [109]. Acute infection with HCMV in immunocompromised patients is often followed by hematologic disorders such as leukocytosis, bone marrow aplasia with thrombocytopenia and leukopenia, and the inhibition of blood cell production in the bone marrow of transplant patients [49]. Stromal cell infection within the bone marrow seems to inhibit hematopoiesis [51].

MCMV also infects and establishes latency in the bone marrow [110, 111]. MCMV infects a broad range of bone marrow cells but induces latency in hematopoietic stem cells, myeloid progenitor cells, monocytes, and DCs and can be reactivated during DC differentiation induced by LPS [110]. During the first week of MCMV infection, a reduction in the number of precursors for granulocyte–monocyte or erythroid lineages is observed in the bone marrow of BALB/c mice [112, 113]. In MCMV-resistant C57BL/6 mice, NK cells prevent hemopoietic dysfunction, whereas NK cells enhance hemopoietic dysfunction in BALB/c mice, indicating that the inflammatory response also contributes to hematopoietic dysfunction [114]. MCMV also inhibits the replenishment of the hematopoietic stem cell pool following sublethal γ-irradiation [115]. MCMV infection causes bone marrow aplasia accompanied by reduced numbers of long-term hematopoietic cells (LT-HSCs), which are required to replenish blood and immune cells upon hematopoietic stress [116]. MCMV infection results in early increases in the levels of IFNα, IL-10, IL-12, CCL2, CCL3, and CCL4 in the bone marrow, and this proinflammatory environment potentially affects LT-HSC function [116]. Both acute (day four p.i.) and nonacute (day 21 p.i.) infections reduced the potential of LT-HSCs to generate immune cells [116]. Fortunately, at four months p.i., LT-HSCs did not exhibit functional deficits. After infection in neonatal mice, NK cell generation in the bone marrow exhibits long-term impairment through a currently unknown mechanism [117]. Finally, following hematopoietic cell transplantation, CMV can inhibit hematopoiesis [51], whereas adoptively transferred antiviral CD8 T cells can control the infection of stromal cells within the bone marrow and allow efficient hematopoiesis in mice [118].

In conclusion, although CMV pathogenesis has been extensively studied in lymphoid organs, especially in the spleen, in mouse models, there is still much to learn. Understanding how CMV manipulates immune responses in lymphoid organs might have practical applications in medicine, especially in oncology and transplant medicine.

## The immune response to cytomegalovirus infection in nonlymphoid organs

Specialized immune responses occur in different tissues to protect against pathogens and maintain tissue integrity [119]. Anatomical localization dictates the tissue-specific immune cell composition, affecting immune cell phenotype, subset differentiation, and function. Additionally, the route of pathogen entry defines the outcome of the immune response in different tissues [119]. For most nonlymphoid organs, the initial immune response starts with the activation of tissue-resident macrophages [119]. Resident macrophages represent a heterogeneous population of cells that switch their phenotype and adapt to different functions in response to inflammatory stimuli [120]. Specialized tissue-resident macrophages, such as Kupffer cells in the liver, alveolar macrophages in the lungs, and histiocytes in intestinal tissue, rapidly react to perturbations in their environment and recruit other immune cells to sites of infection [120]. Although macrophages suppress inflammation mediated by infected monocytes, these cells often represent an important target of infection, produce proinflammatory mediators, and induce robust inflammatory responses. In tissues referred to as “immune-privileged” sites, such as the brain, eye, or testis, resident macrophages ensure tissue homeostasis, preventing strong inflammation to minimize tissue damage and avoid pathology [119, 120]. Different subsets of dendritic cells are present within nonlymphoid tissues, where they produce various cytokines and activate specific NK cell subsets or prime specific T-cell responses. Primed T cells can enter tissue, even in “immune-privileged” organs, and resolve productive infections [121, 122]. Furthermore, CMVs induce tissue-resident memory T (T_RM_) cells in both human and mouse nonlymphoid tissues, where CMVs also establish latency and persistence, facilitating lifelong virus surveillance [123].

### Eye

Multiple cell types necessary for proper eye function, such as retinal cells, keratocytes, and glial and inflammatory cells, can be infected with HCMV [124]. The most common visual system disease associated with CMV infection in immunocompetent persons is anterior uveitis (AU), which is associated with inflammation in the anterior eye chamber [125]. In immunocompromised patients, on the other hand, the most prevalent opportunistic infection caused by HCMV is retinitis, which is characterized by necrosis of the retina caused by the cytopathic effects of the virus [126]. During chronic ocular HCMV infection, CD163^+^ macrophages, CD68^+^ macrophages, and CD3^+^ T-cell infiltrates can be found close to HCMV-positive cells [124]. In addition, high IL-5, IL-6, IL-8 (CXCL8), CCL-2, CCL-4, G-CSF, and TGF-β expression was detected in the ocular aqueous humor of patients infected with HCMV [124]. In AIDS patients, low CD4 and CD8 T-cell numbers are risk factors for the development of HCMV retinitis. In contrast, immunocompromised individuals are not prone to developing AU, suggesting that the immune system contributes to AU development [127]. Conversely, after initiating anti-HIV retroviral therapy, some patients develop immune reconstitution inflammatory syndrome, which is likely caused by reactivated T-cell responses [126]. In addition to infection of immunocompetent and immunocompromised adult individuals, CMV infection of the eye has also been documented in congenital HCMV infection [128].

Several mouse models have been used to investigate CMV infection of the eye, including intraocular injection of the virus [129], the use of mice with a disrupted blood‒retinal barrier [130], or the use of immunocompromised mice [131]. Systemic infection of immunocompetent but CMV-sensitive BALB/c mice with a virulent salivary gland-derived virus (SGV) preparation results in eye infection followed by chronic inflammation [132]. Similar to HCMV, MCMV infects the anterior segment of the eye and spreads throughout the uveal tract; however, it does not infect the retina. MCMV can also be detected in the endothelial and perivascular cells of the iris. Furthermore, ocular MCMV infection resulted in chronic uveitis associated with the retention of virus-specific CD8 T_RM_ cells in the iris and retina. Similar to other organs, the virus can establish latency in the eye following the acute phase of infection and can be reactivated [132]. CMV pathogenesis in the anterior ocular segment was also studied in immunocompetent rats. An association between elevated intraocular pressure (IOP) and immune cell infiltration was observed following RCMV injection into the anterior chamber of the eye [133]. The first peak of IOP was associated with the infiltration of innate immune cells, such as NK cells and antigen-presenting cells. In contrast, the second peak of IOP was caused by the infiltration of adaptive immune cells, such as CD8, CD4, and NKT cells, eight days after infection [133]. Thus, the immune response during both acute and latent CMV infection is a critical factor in the pathogenesis of eye infection.

### Gastrointestinal tract

HCMV infections are common triggers of colitis, gastritis, or enteritis in both immunocompromised individuals and those with inflammatory bowel disease (IBD) (reviewed in [134, 135]), with occasional cases reported for immunocompetent patients, particularly those following primary HCMV infection [136]. Viral inclusions have been detected in endothelial and epithelial cells, fibroblasts, smooth muscle cells, and macrophages [137]. Upon HCMV infection, inflammatory macrophages perform a critical function in initiating or impairing inflammation of the GI mucosa and associated diseases [138, 139]. Following primary infection, HCMV-infected monocytes migrate from the blood to the lamina propria of the GI tract, where they differentiate into inflammatory macrophages, which respond to invading bacteria through robust inflammatory cytokine release [139]. CMV interferes with the TGF-β-mediated inactivation of the NF-κB pathway by inducing the TGF-β antagonist Smad7, preventing the conversion of inflammatory monocytes into inflammatory-anergic intestinal macrophages [139]. Intestinal epithelial cell (IEC) lines, as well as murine and human colonic tissue, express functional receptors for IFNλR that induce the expression of antiviral proteins by activating STAT1 and strongly inhibiting HCMV protein expression after infection [140].

In individuals with AIDS who suffer from severe HCMV disease, the gastrointestinal tract is one of the most commonly infected sites. This involvement often presents as cytomegalic cells and mucosal lesions resembling those observed in inflammatory bowel disease [134, 135]. HCMV plays a significant proinflammatory role as a cofactor in intestinal barrier dysfunction during asymptomatic HIV infection [141]. CMV replicates in the gut of HIV-positive individuals, disrupting the integrity of intestinal epithelial cells, which leads to reduced transepithelial electrical resistance and increased epithelial barrier permeability, with CMV-induced IL-6 playing a role in mediating these effects. Interestingly, early-life HCMV infection has been associated with gut microbial dysbiosis and an increased risk of developing allergic diseases during childhood [142].

Acute MCMV infection in immunocompetent BALB/c mice temporarily alters the ratio of the *Firmicutes* to *Bacteroidetes phyla* in the gut [143]. This dysregulation of the microbiota coincided with high viral titers in the colon, mild pathological changes in the gut architecture, such as crypt hyperplasia, increased levels of colonic proinflammatory cytokines (TNFα, IFNγ, and IL-6), and a leaky intestinal epithelial barrier, consequently increasing intestinal inflammation [143]. Furthermore, gut infection is associated with the generation of intraepithelial MCMV-specific inflationary memory T cells, as shown in C57BL/6 mice [144]. These CD8 T cells express the tissue residency markers CD69 and CD103 and exhibit transcriptional profiles distinct from those of their splenic counterparts, which may be relevant to protection against mucosal infections.

The mechanisms by which CMV induces inflammation in the GI tract are mostly elucidated utilizing mouse models of IBD. MCMV infection was shown to worsen colitis in TCR-α KO mice, as indicated by more severe hyperplasia of epithelial cells in the colon, infiltration of inflammatory cells such as inflammatory macrophages and neutrophils, and crypt loss in these mice compared with uninfected TCR-α KO mice [145]. Moreover, Th1/Th17 and Th2 immune responses were heightened in the colonic mucosa of infected TCR-α KO mice. Most of the MCMV-infected cells in the colonic mucosa were perivascular stromal cells, including PDGFR-β^+^ and CXCL12^+^ pericytes, suggesting that CMV reaches the gut through the blood.

Several studies have shown that MCMV accelerates the development of DSS-induced colitis [146–148]. Mice infected with MCMV exhibited a shortened colon length, increased infiltration of inflammatory cells to the colon, and higher histopathology scores than those treated with mock inoculum [146]. In addition, MCMV infection enhances IL-23 production in the colon, and the administration of anti-IL-23R monoclonal antibodies helps reduce accelerated colonic inflammation in infected mice [146]. However, the development and intensity of MCMV-induced colitis appear to vary based on the mouse strain and the dosage of the virus or DSS, as differences in pathology and virus control exist among various models [149]. Changes in the gut microbiota were also observed in macaques following RhCMV infection [150, 151].

The pancreas is also highly susceptible to infection [152]. In fact, CMV is the most common viral infection affecting the pancreas in transplantation recipients and is associated with significant morbidity and mortality [152]. In a study of 34 fetuses with congenital HCMV (cHCMV) infection that died in utero, infection of the pancreas was observed in all patients [153]. Epithelial acinar cells, duct cells, Langerhans islets, mesenchymal cells, and capillary endothelial cells were all CMV positive in the infected fetal pancreas. An infection in the pancreas is accompanied by focal necrosis and mild to severe CD45^+^ inflammatory infiltrates [153]. Similar findings were observed in another study involving 45 fetuses from women who underwent primary HCMV infection [154]. The viral load in the pancreas was correlated with the level of cerebral damage, with the highest being in infants with severe cerebral damage [154]. A recent study reported HCMV reactivation in the pancreas in the case of fulminant type 1 diabetes mellitus, where infiltration of macrophages and CD4 and CD8 T lymphocytes was greater in HCMV^+^ islets than in uninfected islets [155]. After i.p. MCMV infection of adult mice with SGV-derived virus, mononuclear inflammatory cells infiltrated the pancreas three days p.i., and acinar cells were positive for MCMV antigens [156]. Inflammation was also observed close to the islets of Langerhans. MCMV-induced pancreatitis was more severe in C57BL/6 mice compared with BALB/c mice despite C57BL/6 mice being more resistant to MCMV; however, the underlying mechanism remains unknown. IL-1 and TNFα are produced at the peak of infection in the pancreas, and treatment of mice with the same cytokines reduces the development of acinar necrosis without affecting viral replication or inflammation [156]. Furthermore, MCMV causes insulin resistance and disrupts glucose intolerance in prediabetic mice [157]. Thus, CMV is an important factor to consider in patients with IBD and diabetes, two diseases of modern age with increasing incidence in the general population.

### Lungs

Although rarely occurring in immunocompetent individuals [158], HCMV-associated pneumonitis is the most common clinical presentation of HCMV infection after hematopoietic stem cell transplantation [159]. Solid organ transplant recipients and AIDS patients are also at high risk of developing lung HCMV infection [160, 161], and the lungs are also often affected by congenital and acquired HCMV infection in infants [162]. In rare cases, HCMV infection in neonates can cause pneumonitis, followed by chronic lung disease and fibrosis that can lead to the development of long-lasting bronchopulmonary dysplasia [162]. Interestingly, HCMV pneumonitis is observed at higher rates in neonates who acquire the infection perinatally rather than congenitally probably due to virus inhalation and direct spread to the respiratory system, which cannot occur during infection in utero [76]. In the respiratory tract of immunocompromised individuals, alveolar epithelial cells, mesenchymal cells, DCs and macrophages are susceptible to HCMV infection [137]. Postmortem immunohistochemical analysis of infected fetal tissues revealed strong infection of the alveolar epithelium, as well as endothelial and mesenchymal cells [153]. In children with HCMV infection of the lungs, an inflammatory response characterized by monocyte infiltration was observed [163]. In immunocompromised individuals, impaired CD8 T-cell immunity has been implicated in the progression of HCMV infection of the lung [164]. Upon the resolution of acute infection, T cells remain in the lungs during the latent phase [23].

MCMV infection recapitulates manifestations of HCMV infection in the lungs [165]. In accordance with human cases of HCMV infection, different lung cells are targets of MCMV infection [166, 167], with endothelial cells being the immediate targets of MCMV following i.p. infection [168]. After intranasal inoculation, alveolar macrophages (AMs) are the main target of MCMV infection [169]. MCMV reprograms AMs, causing an attenuated inflammatory response, enhanced MCMV dissemination, and increased susceptibility to secondary bacterial infection [170]. Following immunosuppression by total body irradiation, CD8 T cells are crucial for controlling MCMV infection in the lungs [166]. Furthermore, the depletion of CD4 T cells also increased virus titers in this organ [101], and CD4 T cells secured control when CD8 T cells were depleted [171].

**Fig. 2:**
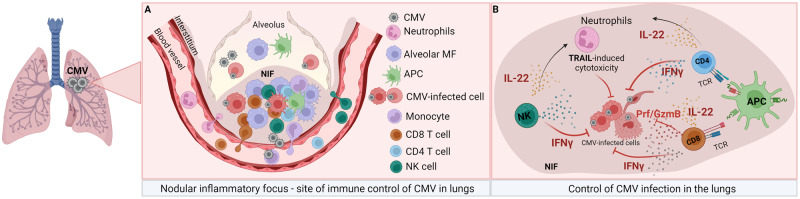
Immune response to CMV infection in the lungs. **A** Nodular inflammatory focus in the lungs. In the lungs, CMV infects alveolar macrophages (AMs), endothelial cells, fibroblast-like cells, and dendritic cells. NIFs form within the infected lung as sites of CMV control and contain different leukocyte populations, such as monocytes, neutrophils, NK cells, and T cells. **B** Control of CMV infection in the lungs. Antigen-presenting cells (APCs) present viral antigens to infiltrating T cells via MHC-I and MHC-II molecules. T cells, mainly CD8 T cells supported by CD4 T cells, produce IFNγ and cytolytic granules to control lung infection. Additionally, NK cells also contribute to virus control in an IFNγ-dependent manner. IL-22, which is produced by NK and T cells, recruits neutrophils at the site of infection. Neutrophils exert their antiviral effects in a TRAIL-dependent manner

Other immune cells have also been recently characterized as important mediators of MCMV control in the lungs. The depletion of alveolar macrophages using clodronate liposomes reduced viral titers in the lungs of neonatal mice [169]. Neutrophils infiltrate the lungs in an IL-22-dependent manner following i.p. MCMV infection, and neutrophil depletion using anti-Ly6G antibodies increases viral loads in the lungs [172]. The antiviral activity of neutrophils in MCMV-infected lungs is TRAIL dependent [172]. MCMV-infected mast cells also protect against MCMV by secreting the chemokine CCL5, which recruits CD8 T cells [173]. Adult and neonatal mice infection is associated with focal clusters of infected cells surrounded by immune cells called “nodular inflammatory foci” (NIFs) [174] (Fig. 2A). The infiltrating immune cells localized close to the MCMV-infected cells in the lungs were mainly CD11b^+^ and/or CD11c^+^ cells, and a lower proportion of T cells and NK cells was observed. The control of viral infection in NIFs is mediated primarily by CD4 and CD8 T cells in an IFNγ-dependent manner [175] (Fig. 2B). NK cells also produce IFNγ and provide some protection in the absence of T cells. Interestingly, the prolonged presence of NIFs is observed in neonatal mice [174], consistent with the reduced ability of the neonatal immune system to control infection. Unconventional γδ T cells also contribute to controlling MCMV infection in the lung, as observed in mice lacking CD8 and CD4 T cells [176]. Although B cells generally do not contribute to the control of acute MCMV infection [102], their role was observed in the lungs of mice lacking the key NK cell receptor NCR1 [62]. Apart from controlling infection, it was recently demonstrated that the immune response to CMV in the lungs induces allergic airway disease [177]. CMV infection activates migratory and conventional DCs, increasing their antigen uptake, recruitment, and presentation and thus initiating allergic airway disease.

Once the infection is controlled in immunocompetent hosts, NIFs disappear, while antigen-specific T cells can be detected during latency in the lung [175]. MCMV-specific CD8 T cells persisting in the lungs are inflationary, KLRG1^+^CD62L^-^ effector memory T cells and are maintained by IL-15 [178, 179]. Following intranasal infection, MCMV primarily infects the lung parenchyma and olfactory epithelium [180]. MCMV infection via the intranasal route, but not the systemic (i.p.) route, generates CD8 T cells with a T_RM_ phenotype in the lungs, which is distinct from that of effector memory cells [181, 182]. Nevertheless, effector memory cells in the lungs generated by systemic infection can relocate into and protect this organ upon antigen rechallenge [182]. Reporter viruses were used to demonstrate that CX3CR1^+^ monocytes or CD11b^+^ myeloid cells are not the source of reactivating MCMV in the lungs [183]. A more recent study showed that the major site of latency depends on the viral route of infection: PDGFRα^+^ fibroblastic cells are the major site following i.n. infection, and endothelial cells are the major site following i.p. infection [184]. Whether the same applies to HCMV infection remains to be determined.

### Liver

Although the liver is a major target of HCMV infection in humans, HCMV-induced hepatitis occurs very rarely in immunocompetent individuals [185], with elevated liver enzymes being one of the signs of subclinical HCMV infection [186]. On the other hand, HCMV-induced hepatitis with elevated liver enzymes is the most common characteristic of liver infection among immunocompromised individuals, especially after liver transplantation [185]. HCMV also infects the liver during cHCMV infection, resulting in hepatitis or cholestatic disease [187]. Analysis of HCMV-induced hepatitis demonstrated that CMV infects hepatocytes, endothelial cells, and Kupffer cells [188, 189]. It is not entirely understood how HCMV infection induces hepatitis, but necrosis of infected cells and the immune response, including continuous cytokine release, represent essential mediators of liver damage [185]. The accumulation of cytotoxic T cells at the site of tissue injury suggests that these cells could mediate liver damage [190]. Moreover, given that the extent of liver damage does not correlate with the extent of viral replication, it appears that the effect of T cells is indirect [8]. However, T cells are also needed to contain HCMV infection. Liver sinusoidal endothelial cells (LSECs) infected ex vivo with HCMV produce CXCL10 and express ICAM-1, which recruits and activates T cells, including regulatory T (Treg) cells, indicating that LSECs can orchestrate the T-cell response [191]. Human NK cells, which are abundant in the liver, are found within the sinusoids or in the liver parenchyma, with a significant proportion displaying a tissue-resident phenotype [192]. A recent study demonstrated that liver-resident NK cells exhibit an altered phenotype in HCMV-infected individuals and possess enhanced antiviral activity in vitro [193]. Importantly, the presence of NKG2C^+^ and CD2^+^ NK cells in the transplanted liver diminishes the incidence of subsequent viremia following organ transplant procedures, thus emphasizing the protective role these cells play against HCMV.

**Fig. 3:**
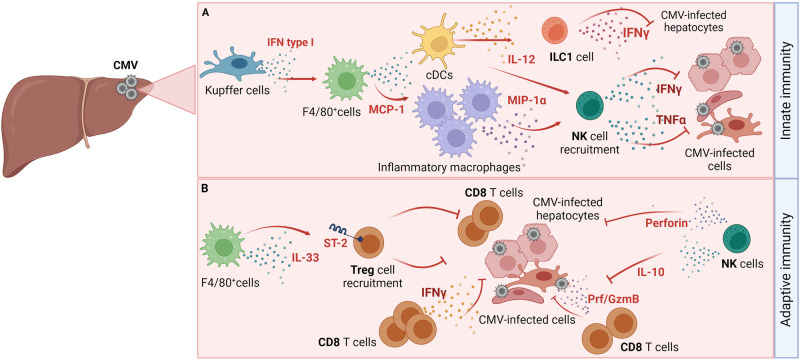
Immune response to CMV infection in the liver. **A** Innate immune response in the liver. Kupffer cells produce type I interferon (IFNαβ) in response to infection, stimulating the production of MCP-1. MCP-1 is critical for recruiting inflammatory macrophages that produce MIP-1α to recruit NK cells to sites of infection. NK cells control the virus in the liver in an IFNγ-dependent manner but can also produce TNFα to sustain virus infection. Conventional dendritic cells (cDCs) produce IL-12 to promote IFNγ production by ILC1s. **B** Adaptive immune response in the liver. CD8 T cells mediate the control of CMV infection in the liver by exhibiting cytotoxic activity. However, the CD8 T cell response to infection in the liver can be exaggerated and can lead to liver pathology. Regulatory T cells (Tregs) and activated NK cells can suppress the pathological response of CD8 T cells to CMV infection in the liver. Tregs (CD4^+^Foxp3^+^ T cells) strongly upregulate the expression of ST-2 receptors and infiltrate the liver in an IL33-dependent manner. IL-33 is produced by F4/80^+^ macrophages. An additional layer of control is exerted by activated NK cells that produce IL-10 and perforin to suppress immunopathology mediated by CD8 T cells in the liver

Similar to that noted in humans, MCMV infection of the liver in mice is characterized by focal inflammation in the parenchyma, mononuclear cell infiltrates [194], and elevated levels of liver enzymes [195] (Fig. 3). Type I IFNs provide critical protection against the virus in the liver and are required for the survival of mice following MCMV infection [196]. Interestingly, the production of IFNα in the liver upon MCMV infection depends on MyD88 but not TLR9 [197]. The first wave of type I IFN in the liver during MCMV infection is produced by Kupffer cells in a STING-dependent manner, whereas the second wave of type I IFN is mediated by TLR/RLR signaling [198] (Fig. 3A). Furthermore, STING was required to restrict the spread of MCMV from liver myeloid cells but not from hepatocytes. In addition to type I IFN, high TNFα levels were also observed at the early stages of MCMV infection, and blocking TNFα diminished the appearance of necrotic foci and reduced the levels of liver enzymes in the sera of infected mice [199]. Early type I IFN induction in the liver promotes MIP-1α production, resulting in the accumulation of NK cells [196], and stimulates MCP-1 (CCL2) production by F4/80^+^ liver leukocytes, which is needed for the accumulation of NK cells and macrophages in the liver [195]. MCP-1 and CCR2 recruit inflammatory macrophages to the liver, where they produce MIP-1α, a molecule critical for the NK-cell-mediated antiviral response. Correspondingly, mice deficient in MCP-1 display an increased viral load in the liver and exhibit elevated liver enzymes (Fig. 3A). Furthermore, depletion of NK cells and neutralization of IFNγ resulted in exacerbated hepatitis outcomes [200–203]. IL-12, a known potent inducer of IFNγ production in NK cells, improves NK cell antiviral responses, and administering low doses of IL-12 decreases the viral titer in the liver and improves hepatitis incidence [202]. Despite monocyte and macrophage infiltration of the sites of infection, inflammatory foci form only when NK cells accumulate in the liver parenchyma [204]. However, some of the effects reported in these previously mentioned studies, might have been mediated by ILC1s, not NK cells; ILC1s are also abundant in the liver and share major phenotypic markers with NK cells [205]. Indeed, it was recently demonstrated that liver ILC1 cells also provide early protection against MCMV infection [205]. ILC1 cells exert their function by early production of IFNγ, which is driven by IL-12 derived from tissue-resident XCR1^+^ cDCs. Liver ILC1s can acquire adaptive features following MCMV infection that promote an enhanced protective response to secondary challenge [206]. Furthermore, it was recently demonstrated that the neonatal liver ILC1 compartment consists of Ly49E^-^ and Ly49E^+^ ILC1s, with Ly49E^+^ ILC1s exhibiting cytotoxic effects and providing protection against MCMV [207].

One of the MCMV strains, known as MCMV v70, replicates in a manner similar to the commonly used K181 MCMV strain but causes rapid and severe hepatitis in BALB/c mice [208]. Hepatitis caused by this strain seems to be T-cell-mediated given that higher frequencies of IFNγ-producing CD8 T cells were associated with more severe disease and depletion of either CD8 or CD4 T cells reduced the severity of MCMV-induced hepatitis. Furthermore, Treg cells prevent CMV-induced liver damage by suppressing the CD8 T cell response [209] (Fig. 3B). Liver Treg cells were activated upon MCMV infection, and high levels of the IL-33 receptor ST2, which is necessary for Treg accumulation in the MCMV-infected liver, were upregulated. IL-33 expression was increased in the liver, and F4/80^+^ macrophages were identified as the cellular source of this cytokine (Fig. 3B). Depletion of Treg cells or infection of ST2-deficient mice led to worsened liver pathology and severe hepatitis without affecting viral load [209]. Thus, Tregs and IL-33 play crucial immunosuppressive roles during MCMV infection in the liver.

Dysregulation of the immune response can also result in severe hepatitis following MCMV infection. IL-10, which is produced predominantly by NK cells, regulates liver inflammation and protects against collateral injury during MCMV infection [210]. MAPKAP kinase 2 (MK2) is critical for regulating cytokine responses during acute MCMV infection, including IL-10 production in an IFNAR-mediated manner [211]. MK2-dependent cytokines, particularly IL-10, prevent the enhanced formation of intrahepatic aggregates of CD11b^+^ mononuclear cells and CD8 T cells expressing the Ki67 proliferation marker observed in IL-10^-/-^ mice. Furthermore, galectin-3 plays an essential role in controlling MCMV in the liver, but it also attenuates liver damage upon infection [212]. TNFα blockade attenuates hepatocyte death and liver inflammation, indicating that TNFα is a significant contributor to liver damage in galectin-3 knockout mice. Perforin deficiency results in severe liver inflammation following MCMV infection [213, 214]. These immunopathological processes are driven by CD8 T cells, as depletion of CD8 T cells in perforin-deficient mice reduced liver lesions and mortality. In mice, liver endothelial cells, along with PDGFRα+ fibroblastic cells, are the major site of MCMV latency and reactivation [184, 215]. However, it is unknown whether lifelong latent CMV infection in the liver has a direct impact on liver health or whether it contributes to the development of other liver diseases.

### Kidneys

The immune control of cytomegalovirus infection in the kidneys has not been extensively studied, but it is known that kidney epithelial and endothelial cells, as well as fibroblasts, can be infected with HCMV [10]. Interestingly, cHCMV infection of the kidneys is not associated with an increased risk of malformations, and kidney failure is rarely associated with cHCMV infection [216]. Typical pathological characteristics were observed in the kidneys of cHCMV patients, with inclusion bodies, both intranuclear and cytoplasmic, detected in the tubular epithelium and cortical tubules of the kidneys at earlier gestational weeks. At later time points p.i. or a later gestational age, inclusion bodies are detected in the epithelial and endothelial cells, and focal inflammation with mononuclear infiltrates also becomes observable [216]. Symptomatic and asymptomatic children excrete HCMV in urine for prolonged periods of up to several years [217]. CMV is also the most common opportunistic pathogen in kidney transplantation [218] and can lead to renal rejection [219]. In CMV-infected renal transplant patients, NK and T cells can protect against infection and prevent transplant rejection [220]. However, the exact mechanisms protecting kidney tissue from CMV-induced pathology have not been elucidated. Kidney infection was not readily observed upon infection of adult mice. Nevertheless, it was observed upon infection of embryonal or neonatal mice [221], where the virus can persist in kidneys for three months following infection [222]. Accordingly, in contrast to infection of adult mice, urinary excretion is a common feature following infection of mouse embryos or neonatal mice [221]. Considering the complications of CMV infection in kidney transplant patients and prolonged shedding of the virus in the urine of children, understanding the immune mechanisms controlling infection in this tissue is of prime interest.

### Adrenal glands

Adrenal glands represent one of the most affected organs in the early stages of HCMV infection in immunocompromised individuals and infants [223, 224]. The noteworthy tendency of CMV to infect adrenal glands is intriguing, considering its indispensable role in the hypothalamic‒pituitary‒adrenal (HPA) axis, which regulates stress responses through the production of glucocorticoids (reviewed in [225]). HCMV can infect both primary adrenocortical cells and adrenocortical cell lines in vitro, inducing noticeable cytopathic changes in cells and stimulating cortisol production via activation of steroidogenesis [226]. Consistent with these findings, several reports have associated HCMV infection with adrenal gland insufficiency, especially in AIDS patients [223] and during cHCMV infection [227]. Further investigations have revealed instances of adrenal involvement in otherwise healthy infants [224] and immunocompetent adults infected with HCMV [228]. Symptoms of adrenal gland insufficiency gradually appear, typically when 80% to 90% of the adrenal cortex is damaged, suggesting that adrenal gland infections in healthy individuals rarely cause severe impairment or symptoms [224]. On the other hand, symptoms that occur late, when the gland is already significantly damaged, make CMV infection potentially highly dangerous in immunosuppressed patients.

The precise dynamics of HCMV entry into the adrenal gland, its clearance, infection rate, its ability to exist in a latent state, and its ability to reactivate HCMV have still not been thoroughly investigated, and most related studies have been performed using MCMV as an infection model. Like HCMV, MCMV readily infects the murine adrenal adenocarcinoma cell line Y1, causing cytopathic changes [229]. MCMV infection of immunocompetent BALB/c mice induces adrenalitis, with the virus replicating in the adrenal gland cortex [78, 230, 231]. Additionally, MCMV-infected mice exhibit an increase in glucocorticoid levels mediated by IL-6; these levels peak at 36 h p.i. and safeguard against TNF-mediated lethality [232, 233]. Thus, the induction of endogenous glucocorticoids protects against pathological effects caused by cytokine responses induced by infection. The complex interplay among hormone production, immune response, and the adrenal gland is further illustrated in experiments using adrenalectomized BALB/c mice, which produce increased amounts of IFNγ, TNFα, IL-6, and IL-12 compared with their nonadrenalectomized counterparts and succumb to low, otherwise well-tolerated doses of MCMV [231, 233]. One possible explanation for the high MCMV mortality in the absence of adrenal glands could be attributed to the adverse effects of cytokine-induced hyperinflammation given that the high sensitivity of adrenalectomized mice to MCMV can be ameliorated using corticosterone replacement therapy [233]. A high viral load in adrenal glands was observed in immunocompromised irradiated BALB/c mice [230] and homozygous nude athymic mice, which lack T cells [234, 235]. In these cases, MCMV replication within the adrenal glands causes progressive focal necrosis of both the adrenal cortex and adrenal medulla, which ultimately results in extensive destruction of the adrenal gland tissue. Importantly, this phenomenon was reversed when T-cell function was restored by the adoptive transfer of naive splenocytes before infection [234], MCMV-specific splenocytes [235, 236], or MCMV-specific CD8 T cells [230]. In fact, CD8 T cells are key players in resolving infection and reducing pathological changes within AGs [230].

Although little is known about the function and phenotype of other immune cells in adrenal glands, some studies have investigated the role of endogenous glucocorticoids and catecholamines in regulating the immune response during MCMV infection. A dysregulated HPA axis has been linked to numerous autoimmune and inflammatory diseases [237] and the immune response to MCMV infection [232, 238, 239]. In this context, the HPA axis modulates the immune response and prevents tissue damage resulting from an excessive immune reaction [233]. Moreover, NK cells devoid of glucocorticoid receptors exhibit increased IFNγ production during MCMV infection [238]. Glucocorticoids upregulate the expression of the known checkpoint target receptor PD-1 in splenic NK cells, which limits excessive NK cell reactions to infection and pathology in the spleen but has minimal effect on virus control. In addition to glucocorticoids, catecholamines also regulate immune responses. Furthermore, two studies showed that the beta-2 adrenergic receptor (β2-AR) appears to have diverging roles during MCMV infection [240, 241]. Indeed, β2-AR signaling is a cell-extrinsic negative regulator of IFNγ production by NK cells at early stages of infection and can have negative effects on virus control [240]. Conversely, cell-intrinsic adrenergic signaling is beneficial at later stages by promoting NK cell expansion and the adaptive response in the spleen [241].

In conclusion, the pathogenesis of CMV and its impact on the adrenal glands, which are vital components of the endocrine system, remain poorly understood. However, CMV infection can disrupt their function, potentially resulting in adrenal insufficiency and severe health complications, particularly in immunocompromised individuals. Therefore, understanding the interplay between CMV infection, the immune response, and adrenal gland function is important for identifying potential therapies that could prevent adrenal insufficiency and alleviate its consequences.

### Salivary glands

One of the major modes of CMV transmission is through shedding in mucosal secretions such as saliva, breast milk, semen, vaginal fluid, and urine, and these fluids are major dissemination sources during both the acute phase and during reactivation from latency. The latent virus reactivates in nearly all seropositive women during lactation, and CMV-infected breast milk not only becomes a significant source of primary HCMV infections via the oropharyngeal route but also poses a severe threat to preterm newborns (reviewed in [242]). The secretion of HCMV in the saliva is of high epidemiological significance because it can persist for years after the initial infection during childhood (reviewed in [243]). The long-term shedding of the virus in saliva during HCMV infection in children could be caused by long-lasting deficiency of CD4 T cells in children [244].

Salivary gland (SG) MCMV preparations, which have historically been used as a source of the virus, are significantly more virulent than tissue culture-derived viruses. Attenuation of SG-derived virus occurs after a single passage in mouse embryonic fibroblasts [245] and can also be reversed after a single in vivo passage and isolation from salivary glands [246]. Following i.v. or i.p. infection, MCMV becomes detectable in SGs approximately five days p.i., reaches peak titers between 14 and 21 days p.i. and then persists in SGs for weeks or even months [247]. The kinetics of virus replication in the SG of both SG viruses and tissue culture-derived viruses are similar, and infected monocytes mediate entry into the salivary glands [78]. Following intranasal infection, MCMV-infected CD11c^+^ DCs exit the lungs and disseminate the virus through LNs to SGs, where the virus becomes detectable by plaque assay on day six p.i [80]. The importance of SG infection is best exemplified by numerous MCMV genes dedicated to enhancing infection of this organ, such as the virally encoded chemokine MCK-2 [31, 248–250], M116.1, which is required for mononuclear phagocyte infection [33], and M78, which facilitates MHC-II degradation and thus protects antigen-presenting cells (APCs) from CD4 T-cell control [251]. In addition to those already listed, MCMV-encoded sgg1 and M33 are also required for successful replication in SGs; however, the mechanism for sgg1 and M33 involvement remains unclear [82, 252–254].

**Fig. 4:**
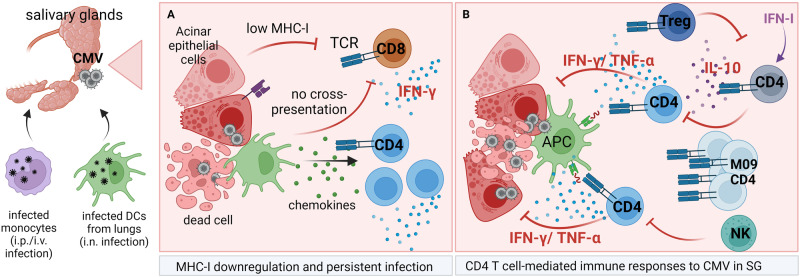
Immune response to CMV infection in the salivary glands. CMV reaches the salivary gland via antigen-presenting cells: monocytes following i.p. or i.v. infection or DCs following i.n. infection. Numerous immune cells are recruited to CMV-infected salivary glands; however, unlike in any other organ, the CD4 T cells control the infection. **A** CD8 T cells are recruited to infected SGs and secrete IFNγ; however, they are unable to recognize infected cells because acinar endothelial cells express very low levels of MHC-I and local APCs are unable to cross-present antigens. CD4 and CD8 T cells are likely recruited by proinflammatory cytokines secreted by APCs and infected epithelial acinar cells. **B** CD4 T-cell immune responses in the SG. In addition to IFNγ/TNFα-secreting CD4 T cells, which control infection, IL-10-secreting CD4 T cells are recruited by IFN-I with delayed kinetics compared with IFNγ/TNFα-secreting CD4 T cells and cause virus persistence. IL-10 CD4 T cells are subsequently controlled by Tregs. Activated CD4 T cells are also regulated by NK cells, which limit their number and prevent immune-mediated pathology (e.g., Sjogren’s syndrome). At later stages of infection, M09-specific CD4 T cells accumulate in SGs and are required for virus clearance

Acinar glandular epithelial cells, a highly abundant cell type in SGs, have been identified as the main cell type supporting infection (Fig. 4) [255]. These cells contain multiple nucleocapsids in cytoplasmic inclusion bodies during the persistent phase of infection [101]. A more recent study utilized in vitro infection of primary human acinar epithelial cells in monolayers or salispheres, globules of epithelial cells that possess primitive structures akin to salivary glands, using several HCMV strains. In this study, HCMV seemed to be trapped in acinar epithelial cells, neither spreading through the supernatant nor in a cell-to-cell manner, whereas the virus appeared to have progressed through the immediate early, early, and late stages of infection [256]. Compared with fibroblasts or endothelial cells, infected acinar epithelial cells exhibit excessive cell death. The authors hypothesized that the acinar epithelial cells died and detached before cell-to-cell spread could occur. Moreover, low levels of virus replication could be detected in acinar epithelial cells for extended periods in vitro after infection with a low dose of virus. A similar observation of the poor ability of CMVs to spread from one infected acinar epithelial cell to another was made in a mouse model of CMV infection following i.p. infection of NSG mice using high doses of the K181 MCMV strain [254]. The virus might spread in an interacinar fashion, be secreted into the duct, and thus be secreted into the saliva.

Unlike in other organs, in SGs, CD8 T cells are dispensable for virus control (Fig. 4A) [101]. One study utilized MCMV lacking MHC-I immunoevasins (m04, m06, and m152) and demonstrated that such viruses are efficiently controlled in the salivary gland by CD8 T cells [257]. However, this study used an i.p. route of infection during which the virus reaches the spleen, liver, and LNs before SGs, where CD8 T cells control virus replication; thus, a lower amount of virus reaches SGs [46]. Another study reported that the levels of MHC-I molecules in acinar epithelial cells were undetectable in CMV-infected cells [258]. A recent study demonstrated strong downregulation of MHC-I in some virus-positive cells [259]. Furthermore, APCs from the salivary glands cannot cross-present engulfed antigens to CD8 T cells (Fig. 4A) [258].

Instead of CD8 T cells, CD4 T cells are critical for controlling viral infection in SGs by secreting TNFα and IFNγ, which act on nonhematopoietic cells (Fig. 4B) [101, 258, 260, 261]. Viruses lacking M78, an immune factor that degrades MHC-II in infected cells, exhibit a diminished ability to colonize SGs following i.n. infection, and this effect can be partially rescued with CD4 T-cell depletion or using mice deficient in MHC-II [251]. CD4 T cells infiltrate SGs approximately one week after systemic infection in mice, expand and reach peak numbers by week 2, and then contract and persist in SGs for an extended period. Infiltrating cells display the effector Th1 phenotype during the acute phase and memory phenotype at later time points (reviewed in [262]), and these cells require in situ antigen stimulation for their generation [259]. Adoptive transfer experiments in irradiated mice demonstrated that virus-specific CD4 T cells can protect against MCMV, not only in SGs but also in other organs. The M09-specific late-arising CD4 T-cell subset was recently shown to resolve persistent infection in C57BL/6 mice (Fig. 4B) [263]. Unlike CD8 T cells and HCMV-specific CD4 T cells, MCMV-specific CD4 T cells do not seem to undergo memory inflation [264]. Active virus replication is eventually resolved in SGs even without CD4 T cells, but the process may last up to 400 days, whereas mice deficient in MHC-II still exhibit active virus replication in SGs by day 400 [258].

In addition to IFNγ secretion, some CD4 T cells present in SGs, such as Th1 cells, also secrete IL-10 during MCMV infection (Fig. 4B). These CD4 T cells secrete IL-10 in response to IL-27, which is induced by type I IFNs secreted in response to CMV infection. CD4 T-cell-secreted IL-10 limits immune responses to CMV and facilitates the long, acute virus replication characteristics of SG [265–267]. Interestingly, the inability of CD4 T cells to secrete IL-10 does not lead to the induction of autoimmunity, most notably Sjogren’s syndrome [266]. Treg cells in SGs are also present and, interestingly, prevent virus reactivation by suppressing the activity of IL-10-secreting CD4 T cells (Fig. 4B) [268].

Although dispensable for virus control, CD8 T cells secrete even more IFNγ than CD4 T cells, migrate into MCMV-infected SGs [247] and are functional even without the help of CD4 T cells [258]. A substantial pool of CD8 T_RM_ cells specific for inflationary epitopes of MCMV is recruited to SGs from the periphery, and their generation does not depend on the presence of local antigens [259, 269]. Virus-specific CD8 T cells from the periphery are proposed to be the source of SG T_RM_ cells. Although the recruitment of antigen-stimulated CD8 T cells from the periphery is inefficient, occasional antigenic boosts from sporadic reactivation events in the host eventually result in significant numbers of virus-specific CD8 T cells that reach SGs from the periphery and become T_RM_ cells. These CD8 T_RM_ cells then protect against localized reactivation and challenge infection in, e.g., fibroblasts [259], although these cells express high levels of PD-1 and other exhaustion markers [270].

NK cells are also present in SGs, and more NK cells are recruited to SGs during MCMV infection. However, in contrast to other organs and similar to CD8 T cells, the capacity of these cells to control virus replication is limited both in vitro and in vivo. The inability of NK cells to control the virus might be caused by the low amounts of MHC-I and high amounts of KLRG1 ligands in SGs and lower levels of NKp46 expressed by SG NK cells [271]. Despite lower NK cell activity in SGs, NK cell control is required to protect the host from the destruction of SG function [272] by eliminating activated CD4 T cells through TRAIL-induced apoptosis, also resulting in prolonged viral shedding (Fig. 4B) [273]. A recent study examined the phenotype of NKs and ILC1s in SGs in greater detail and identified ILC1s, tissue-resident NKs, and classical NKs based on a combination of the following markers: Eomes, Ly6C, CXCR6, CD49a, CD49b, CD127, CD11b, and KLRG1 [274]. Following MCMV infection, classical NK cells (Eomes^+^CD49b^+^) are recruited from the periphery, enter the SG parenchyma, and become long-lived NK resident memory cells (NKRMs) with a distinct phenotype (Ly6C^+^, CD49a^+^, CXCR6^+^, CD127^+^, CD11b^−^, KLRG1^−^). The recruitment of these cells to SGs relies on CX3CR1 signaling [274]. Interestingly, these cells formed most efficiently in the absence of the m157-Ly49H interaction. In C57BL/6 mice, a distinct NK cell population formed following CMV infection; however, this population lacked the markers of NKRM observed in BALB/c mice. These NKRM cells kill CD4 T cells in a TRAIL-dependent manner [274]. Finally, NK cells also limit the hematopoietic cell-mediated dissemination of MCMV into SGs after intranasal infection [275]. Accordingly, NK-sensitive MCMV lacking the m15 gene region is attenuated in SGs, and fewer viruses are present in the saliva. Whether this phenotype results from lower seeding of SGs or diminished replication capacity within SGs is unknown [276].

The salivary gland represents an intriguing organ in the CMV field given its importance in virus host-to-host spread and unusual immune responses. Although CMV pathogenesis in SGs has been a research subject for decades, many open questions remain.

### Reproductive organs

In contrast with the abundant data on CMV infection in the placenta (discussed later) and uterus during gestation, comparatively little data are available about CMV infection in the remainder of the female reproductive system. HCMV can be detected in the vaginal secretions of pregnant and nonpregnant women, indicating that the virus replicates somewhere in the reproductive tract; however, the exact tissue source of the virus is unknown [277–279]. One case report demonstrated CMV reactivation in a uterus transplanted from a CMV-positive donor to a CMV-negative recipient [280]. In that case, HCMV was detected in blood and urine with qPCR but not in cervical biopsies using IHC. The infection was asymptomatic, and the patient delivered a full-term healthy child despite CMV reactivation. Several case reports have described CMV-induced oophoritis, mostly in immunosuppressed, postmenopausal women (latest report and review [281]). CMV was recently detected in ovarian cancer patients and is considered to be a negative prognostic factor [282, 283].

Surprisingly, there are even fewer data from animal studies. In addition to an earlier study in which the MCMV genome was detected in the ovarian stroma of newborn mice [284], our laboratory performed a comprehensive analysis of CMV infection in the ovary during various physiological states: regular estrus, pregnancy, and hormonally induced superovulation [285]. The ovaries were highly susceptible to infection regardless of the hormonal state of the female mice, with the virus detectable on day one post i.v. or i.p. infection and reaching titers comparable to those of the spleen, an organ much larger than the ovary. A major finding was the strict exclusion of the virus from ovarian follicles, whereas the remainder of the ovaries, especially the corpora lutea, were strongly infected. Strong infection of the CL resulted in a dramatic decrease in the serum progesterone concentration and reduced successful pregnancy outcomes. In addition to microanatomical barriers, such as gap junctions, and the absence of capillaries in follicles, innate immune cells (NKs and macrophages) and, most importantly, high NF-κB levels present in ovarian follicles at steady state that mediate immediate type I IFN signaling following infection prevent the virus from reaching the follicles. NKp46^+^ cells (classical NK cells and ILC1s), as well as macrophages, were present in both uninfected and MCMV-infected ovaries and exhibited an activated phenotype (production of IFNγ and granzyme B by NKp46^+^ cells; induction of F4/80 in macrophages) following infection. Mice depleted of or genetically lacking either cell subset exhibited an increased incidence of infection in the follicles.

Viral infections of the testes, in general, have been investigated as a potential cause of infertility based on various mechanisms, such as direct interference with spermatogenesis leading to dysfunction of the sperm, alterations in inflammatory properties of genital secretions, and activation of immune responses that lead to the formation of antibodies against sperm [286]. HCMV DNA can be detected in the seminal tracts of fertile or infertile men, with rates ranging from 8% to 65% [287, 288]. In this context, an ongoing debate persists regarding the influence of HCMV on critical parameters of sperm quality, such as morphology, motility, and concentration. Most studies found no impact of CMV infection on male fertility [287–289], whereas some studies correlated sperm quality with virus titer in the sperm [290, 291]. One study demonstrated that CMV can infect spermatozoa in various stages of development; however, active virus replication was not detected in mature spermatozoa [292]; thus, sexual transmission does not appear to be a significant mode of virus transmission [289]. Latent MCMV can also be detected in the testes of mice and can replicate in male germ cells, including interstitial Leydig cells, where it only temporarily impacts spermatogenesis [293, 294]. A more recent study revealed CMV-induced damage to peritubular cells in the testes and altered spermatogenesis; however, infectious virus was not detected in germ or Sertoli cells. The males undergoing acute infection did not transmit the virus sexually to females or to the offspring, confirming that mature spermatozoa do not carry the infectious virus [295]. Tissue-specific immune responses in the testes during CMV infection have not been investigated.

## Immune response in tissues during congenital cytomegalovirus infection: placenta, brain, and inner ear

Similar to other members of the TORCH (toxoplasma, other agents, rubella, cytomegalovirus, and herpes simplex) pathogen group, HCMV can be transmitted from the mother to the developing embryo/fetus through the placenta. HCMV infection is the most common viral congenital infection, affecting 0.2–2.0% of all newborns worldwide [296, 297]. cHCMV infection is an important cause of neurological diseases, including neurodevelopmental disorders, brain injury, mental retardation, and sensorineural hearing loss (SNHL) [298, 299]. The incidence of vertical transmission increases with gestational age; however, severe consequences are dominant if the infection occurs in the first trimester [300]. Among HCMV-infected newborns, approximately 10% are symptomatic at birth, and the majority exhibit long-term neurological sequelae. Among asymptomatic newborns, approximately 10% will develop cHCMV-associated consequences later in life, most notably SNHL. It remains unclear whether SNHL develops due to viral replication or damage caused by the host immune response [301, 302]. Similarly, uncontrolled viral replication, immune-mediated damage, and fetal hypoxia due to placental insufficiency are considered to be the underlying causes of brain injury induced by cHCMV infection [154].

The rates of intrauterine CMV transmission can be reduced by preexisting maternal immunity to CMV, most notably antiviral antibodies. Early antibody responses to the pentameric complex of HCMV correlate with protection against intrauterine transmission of the virus [303]. Furthermore, women with higher avidity antiviral antibodies do not transmit the virus to the fetus, whereas women with primary infection do [304, 305]. However, these findings have been challenged by recent studies suggesting that no differences in the incidence of clinically apparent cHCMV infection exist in infants born to immune versus nonimmune women (reviewed in [306]). Thus, understanding the extent to which preexisting adaptive immunity, including antibodies, reduces the transmission of the virus to the fetus and decreases the severity of the disease requires additional studies.

### Placenta

The placenta is a temporary organ of dual origin: the maternal portion or decidua and the fetal portion, which consists of the chorion and chorionic villi. Most of our knowledge on HCMV transmission and related immune responses during pregnancy relies on investigations of maternal peripheral blood immune responses (reviewed in [307]). Given that the placenta is intensely perfused by maternal blood, analysis of blood from pregnant women can provide relevant information. For instance, both the intensity of placental infection and the grade of infection-associated placental pathology are inversely correlated with the serum levels of anti-HCMV neutralizing antibodies (reviewed in [307]). Interestingly, treatment of pregnant women with hyperimmune serum did not reduce the viral load or the extent of damage to the placenta [308]. These seemingly contradictory data are potentially explained by the abundant presence of neonatal Fc receptors (FcRn) in the syncytiotrophoblast. Normally, FcRn receptors transfer maternal IgGs across the placenta to provide passive immunity to the fetus. One study demonstrated that FcRns can also transfer HCMV virions bound with neutralizing antibodies across syncytiotrophoblasts to cytotrophoblasts in a process known as virion transcytosis [309]. In patients with moderate to low neutralizing anti-HCMV IgG titers, the virus was transferred across syncytiotrophoblasts and could replicate in cytotrophoblasts. In addition to antibodies, the proliferation of HCMV-specific T cells in the blood and the ability of CD4 T cells to secrete IL-2 are also correlated with protection against vertical transmission of HCMV [310, 311]. Based on the effector-memory phenotype of T cells found in the decidua, it is hypothesized that many T cells are likely recruited from the blood [312].

CMV can successfully infect different decidual cells, especially endothelial cells, as well as trophoblasts, the fetal portion of the placenta, eliciting a strong proinflammatory response that can damage the placenta itself [313], and the same phenomenon was recapitulated in vitro using trophoblast and decidual organoids [314]. Moreover, decidual cells are considered to serve as reservoirs of infectious viruses during pregnancy. Interestingly, decidual cells also upregulate apolipoprotein B editing catalytic subunit-like 3 (APOBEC3 [A3]), a family of cytidine deaminases that catalyze the deamination of cytidines to uridines, resulting in the accumulation of HCMV genome mutations [315]. Trophoblast cells that comprise the fetal portion of the placenta have been consistently demonstrated to be more resistant to infection, both in ex vivo and in vitro models. In a recent study utilizing trophoblast and decidual organoids, distinct differences in response to poly I:C and HCMV, which were previously observed in placental explants, were identified [316]. Decidual organoids secreted more proinflammatory cytokines and chemokines (CXCL10, IL-6, MIP-1α, and MIP-1β) as well as type III IFNs, whereas trophoblast organoids secreted PTX3, IL-8 and IL-11. Although organoids represent somewhat artificial models, this study revealed differences in immune responses to CMV between decidual and trophoblast cells, which may explain differences in susceptibility and permissivity to CMV.

Numerous and various immune cell types present in the uterus and decidua prevent the transmission of the virus from maternal to fetal tissues. In general, decidual NK cells are the most numerous cell type, followed by T cells and macrophages (reviewed in [317]). Nonetheless, changes in decidual and placental tissues during pregnancy are closely paralleled by changes in the composition and phenotype of immune cells. For instance, decidual NK cells are more abundant and display greater cytotoxicity toward CMV during early pregnancy. This phenomenon could explain why vertical virus transmission is more likely during the third trimester of gestation [318]. NK cells can also migrate toward infected cells in the fetal portion of the placenta, and both NK and T cells present in the placenta express higher levels of granulysin than their peripheral blood counterparts [319]. In another study with a somewhat limited sample size, virus-specific T cells of maternal origin were also found in placental villi. However, the exact subtype of the detected T cells (cytotoxic, helper, or regulatory) was not determined [320]. Recently, it was shown that the first-trimester decidual tissues obtained from HCMV seropositive and seronegative patients contained an abundance of immune cells, predominantly decidual NK cells (CD56^bright^), followed by T cells, which are primarily of the effector memory phenotype [321]. Additionally, decidua from seropositive patients contained increased IFNγ levels and were more resistant to HCMV infection in vitro. The sources of IFNγ were CD4 and CD8 T cells, and blocking T-cell recognition of infected cells with pan-HLA-ABC antibodies prevented the induction of IFNγ secretion and abrogated virus control. Furthermore, the majority of T cells in the seropositive decidua were specific for the immunodominant HCMV protein pp65 [321].

Fetal FcRn and FcyIIRA are also expressed by macrophages in the decidua, whereas the chorion contains macrophages known as Hofbauer cells. Macrophages play dual roles in CMV pathogenesis, and the placenta is no exception. Following Fc-mediated transcytosis of HCMV virions from syncytiotrophoblasts to cytotrophoblasts, the virus can also infect Hofbauer cells (reviewed in [322]). Infection with other pathogens can cause reactivation of the HCMV present in decidual macrophages and Hofbauer cells (reviewed in [322]). Similarly, HCMV-infected Hofbauer cells were more susceptible to HIV-1 infection due to CCR5 and CD80 upregulation in infected and bystander cells and the suppression of STAT2-mediated type I IFN responses when infected in vitro [323]. Finally, PD-L1 is upregulated in HCMV-infected Hofbauer cells, with a potentially negative impact on T-cell responses [324].

Although animal models for the investigation of placenta-specific responses to CMV infection exist, they are not utilized as frequently as models for the investigation of CMV infection in other organs or tissues, mainly because (1) the human placenta is readily available and (2) differences in placental structure and virus transmissivity make direct comparisons between animal models and humans challenging. Even in models resembling human placental biology, such as the guinea pig CMV model, the lack of transgenic animals and viral mutants hinders investigations. Recently, a model of nonhuman primates has been used successfully; however, its usage is limited by high costs and ethical concerns [314].

### Central nervous system

**Fig. 5:**
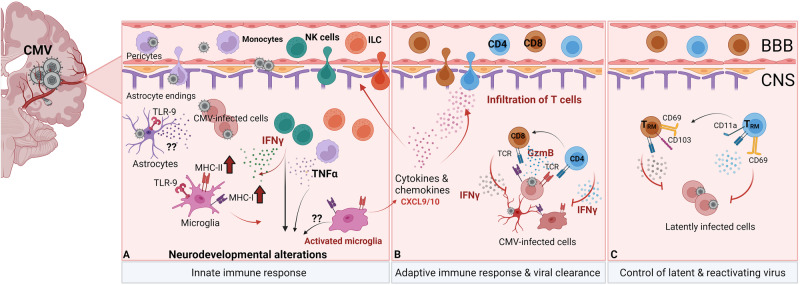
Immune response to congenital CMV infection in the brain. **A** Innate immune response. CMV crosses the blood‒brain barrier (BBB) in a cell-free or cell-associated manner. CMV can infect various resident cells in the CNS, with astrocytes reported as a major infected cell population. Infection results in the production of various proinflammatory cytokines and chemokines that promote microglia activation and the recruitment of peripheral immune cells to the CNS. Monocytes, macrophages, NK cells, and ILCs are the first cell populations to infiltrate the infected CNS. Monocytes and NK cells produce TNFα and IFNγ, respectively, and promote microglial activation. **B** Adaptive immune response and viral clearance. Activated microglia induce MHC-I and MHC-II and produce chemokines, mainly CXCL9 and CXCL10, to attract T cells to the inflamed CNS. CD4 and CD8 T cells infiltrate the CNS, recognize infected cells in an MHC-I- and MHC-II-dependent manner, and control infection by producing IFNγ and granzyme B. **C** Control of latent and reactivating viruses. After productive infection in the CNS is resolved, the virus establishes a latent infection. T cells are tissue-resident memory T (T_RM_) cells that express CD69 and CD103 (CD8 T_RM_) as well as CD69 and CD11a (CD4 T_RM_) and are essential for the control of latent and reactivating viruses in the brain

Upon infection, cytomegalovirus does not penetrate the brain of immunocompetent adult humans or mice [325], but it can infect the CNS during severe immunodeficiency, such as that noted in AIDS patients [326]. In contrast, the virus readily migrates into and infects the brain during cHCMV infection (Fig. 5). It is believed that the increased susceptibility of the brain to infection during the congenital period arises partly from the increased permeability of the developing blood‒brain barrier and the inefficient immune control mediated by the immature immune system. Indeed, numerous mechanisms of immune control are impaired early in life, affecting the response to CMV infection [327, 328]. These include significant differences in T and B cell repertoires [327, 329], a bias of fetal T cells toward the Th2 response [330], and the hyporesponsiveness of NK cells [117, 331]. However, the immune mechanisms that manage and control congenital CMV infection, as well as the reason why congenital infection causes devastating neurological outcomes in only a proportion of cases, remain largely unknown.

The exact route by which CMV spreads from the periphery to the developing CNS is not entirely known. Nonetheless, it is believed that the virus can infiltrate the CNS in a cell-free form (by directly infecting peripheral neurons or cells of the blood‒brain barrier) or in a cell-associated form (via infected brain-infiltrating mononuclear cells) [332, 333] (Fig. 5A). Moreover, CMV infection of the CNS appears to be opportunistic and accidental because it is seemingly not advantageous for the virus. Once in the CNS tissue, CMV can infect all brain resident cells to a certain degree. However, neural stem precursor cells (NSPCs) and astrocytes appear to be the principal targets of infection [334, 335], and the ability of HCMV to infect NSPCs is thought to be a contributing factor leading to abnormal brain development [336]. How the immune system combats infection in different brain cell types currently remains unclear. Innate pattern recognition receptors usually initiate the immune response, and TLR9, a primary sensor for CMV, can be expressed by cells within the CNS, most notably astrocytes and microglia [337]. Activation of microglial nodules, including myeloid cells, NK cells, and CD3^+^ and CD8^+^ lymphocytes [299, 338], which are usually not found in the healthy brain (Fig. 5A, B), was observed in the fetal brains of patients with cHCMV infection and the infiltration of peripheral immune cells. Similar findings were observed upon histopathological analysis of the brains of autopsied infants infected with HCMV [339], providing evidence that an inflammatory response accompanies acute brain infection. Despite their limited numbers, the studies mentioned above have provided valuable insights into immune responses following HCMV infection of the brain. However, due to the limitations and difficulties in studying the progression of CMV infection, the latent phase of HCMV infection in the human brain remains largely unexplored. In a study employing primitive prerosette neural stem cells (pNSCs), HCMV genomes were detected one month after primary infection of these cells with HCMV [340]. Furthermore, the maintenance of viral DNA in the majority of the pNSCs was not attributable to low-level persistent infection, and the functional virions could be reactivated by the differentiation of pNSCs to neurons, suggesting that neural cells might act as a reservoir of the latent virus within the CNS [340]. However, despite the undeniable wealth of data that such studies can provide, they are limited by the lack of complexity and interactions that cells and organoids present in tissue culture would otherwise establish in the context of the entire organism.

Several experimental models, including primates (rhesus macaques), guinea pigs, rats, and mice, have been developed to investigate congenital CMV infection in the brain [341]. Of these, the most commonly used are mouse models of congenital CMV infection, which have provided numerous insights into the mechanisms of immune surveillance and virus control in the brain [24, 335, 342]. One of the limitations of using mouse models to study immune responses to CMV in the CNS is the inability of MCMV to cross the placenta. To circumvent this obstacle and simulate transplacental infection of the offspring, investigators either administer the virus directly to individual fetuses in utero or infect newborn mice. The latter approach is particularly suitable for investigating the effects and immune responses to congenital CMV infection given that the CNS of a newborn mouse is in a developmental stage that generally corresponds to the developmental stage of the brain in the mid- to late second-trimester human fetus [343]. Accordingly, perinatal MCMV infection in mice recapitulates many aspects of cHCMV infection in the brain [344]. Upon i.p. infection of newborn mice, MCMV first replicates in peripheral organs and then spreads to the CNS around day seven p.i., which recapitulates the natural route of CMV dissemination. Furthermore, a strong inflammatory response is induced in the CNS of MCMV-infected newborn mice, which leads to the activation of an early cellular immune response [344]. Infection induces the activation of resident microglia and macrophages, astrocytosis, and infiltration of CD45^hi^ leukocytes in the brain [24, 345]. However, the mechanisms initiating the inflammatory response in the brain upon MCMV infection are poorly understood. Brain infection results in microglial activation, as shown by the upregulation of markers such as MHC-I and MHC-II, proliferation, and overall changes in the patterns of microglial gene expression [345–347] (Fig. 5A). NKp46^+^ NK and ILC1 cells infiltrate the infected brain of newborn mice in a CXCR3-dependent manner, with microglia producing the chemokines CXCL9/CXCL10 [347]. However, mobilized NKp46^+^ cells were unable to control MCMV infection (Fig. 5A). However, CD4 and CD8 T cell infiltration, which peaks at approximately 10−12 p.i., is needed to resolve productive infection in the brain [345, 348] (Fig. 5B). Although the infection in the CNS is efficiently cleared by T cells, the virus remains latent in the brain and can undergo reactivation [348, 349]. Both CD4 and CD8 T cells are retained for a lifetime as brain T_RM_ cells, expressing the prototypical tissue residency markers CD11a, CD69, and CD103 and providing control against latent/reactivating viruses [349] (Fig. 5C). CD4 T cells are also needed to establish and maintain a pool of CD8 T_RM_ cells during latency [348]. B cell responses and their involvement in brain infection have not been studied. However, passive immunization with immune serum or MCMV-specific monoclonal antibodies reduces virus replication and virus-induced pathology in the CNS of MCMV-infected newborn mice [350].

Several studies have investigated immune responses in the brains of adult mice following intracranial injection of MCMV. These experimental findings showed that T cell-derived IFNγ mediates MHC-II expression in microglia [351]. Moreover, Treg cells also infiltrate the brain and attenuate the immune response. Treg cell depletion leads to an enhanced CD4 and CD8 T-cell response and microglial activation, causing a reduction in the cognitive functions of mice [352, 353]. Furthermore, antibody-producing B cells infiltrate the brain early after intracranial MCMV infection, persist during latency, and appear to play a role in restricting viral reactivation [354]. In addition, B cell-deficient mice displayed enhanced microglial activation, reduced numbers of Treg cells, and increased numbers of CD8 T cells [355], suggesting that B cells, in addition to preventing viral reactivation, also constrain the excessive immune response to CMV infection to control neuroinflammation mediated by other immune cell types. However, given that intracranial MCMV inoculation is not a natural route of infection, it circumvents prior activation of the peripheral immune response and results in a damaged blood‒brain barrier and damage-induced inflammation.

In addition to controlling infection, the host inflammatory response is involved in pathological processes in the brain, as demonstrated by an experiment in which the application of glucocorticoids reduced neuroinflammation and infection-mediated CNS pathology in newborn mice with no impact on the control of the virus in any of the organs tested [346]. TNFα secreted by inflammatory monocytes enhances the inflammatory response and infiltration of peripheral myeloid cells into the CNS, and blocking TNFα with neutralizing antibodies abolishes neurodevelopmental abnormalities [356]. Similarly, IFNγ secreted by infiltrating ILC1s and NK cells causes neurodevelopmental delay, and blocking IFNγ with neutralizing antibodies abolishes these neurodevelopmental abnormalities [347]. Furthermore, IFNγ activates microglia and acts directly on neurons to cause neurodevelopmental delay [347]. How TNFα and IFNγ activities are intertwined during CNS infection remains unresolved. However, a plausible explanation could be that TNFα acts on NK cells and contributes to IFNγ production or that IFNγ and TNFα exert synergistic effects. Both TNFα and IFNγ exhibit antiviral effects. However, blockage of TNFα and IFNγ using antibodies did not alter viral titers in infected neonatal mice [347, 356]. Infiltrating T cells also seem to contribute to pathogenesis in the brain following MCMV infection in neonatal mice during the neonatal period [357]. Although MCMV was not detected in the brain following infection in this study, infection resulted in behavioral deficits and reduced social behavior and cognition in adolescent mice [357]. Mechanistically, infection results in microglial activation, leading to increased phagocytic activity and loss of excitatory synapses in the hippocampus. Interestingly, only male mice were affected by infection [357], but the mechanisms underlying sex-dependent functional aberrations remain undetermined.

### Inner ear

SNHL, the most common sequela of cHCMV infection, seems to originate from reduced numbers of spiral ganglion neurons (SGNs) [302, 358]. SNHL can develop months after birth and progress, suggesting that ongoing virus replication could be the underlying cause [359, 360]. In CMV-infected fetuses, the *stria vascularis* and vestibule are predominantly infected, whereas the cells in the organ of Corti are less often infected [301, 302]. Infection is associated with inflammatory responses, including the infiltration of CD8 T cells, macrophages, and B cells. In the spiral ganglia and *stria vascularis* of the cochlea of the inner ear, viral antigens were found close to CD3^+^ infiltrating cells, most of which expressed granzyme B [302]. Lesions displaying cytopathic effects and inflammation are mostly located in epithelial cells bordering the endolymphatic compartment [301]. Furthermore, analysis of samples obtained from older children revealed the presence of calcifications and fibrosis [361].

Guinea pigs and mice were used to experimentally model HCMV infection of the inner ear in vivo. The mouse model recapitulates the major characteristics of human infection in the inner ear and hearing loss [362]. Similar to cHCMV infection in the inner ear [301], following i.p. MCMV infection of newborn mice, virus-infected cells were detected in the spiral ganglia and *stria vascularis* accompanied by mononuclear cell infiltrates [362]. SNHL seems to stem from reduced numbers of spiral ganglion neurons (SGNs) in mice [358]. SGNs receive input from cochlear hair cells, transmitting signals to the auditory cortex. Increased levels of interferon-stimulating genes (ISGs), such as *IFIT1*, the proinflammatory cytokine TNFα, and chemokines, were detected in the cochlea. Interestingly, upon decreasing cochlear inflammation with corticosteroid treatment, SGNs and auditory function in MCMV-infected mice were preserved, suggesting that virus-induced cochlear inflammation contributes to cochlear histopathology and altered auditory function [358]. An intracerebral MCMV infection of neonatal mice resulted in infection-induced apoptosis of SGNs, which was associated with the production of reactive oxygen species (ROS), the proinflammatory cytokines IL-1β and IL-18, and ROS/NLRP3-associated inflammasome activation, all of which contribute to hearing loss [363]. Furthermore, T cell infiltration and TNFα, IFNγ, iNOS, and IL-6 expression were observed [364]. Another study utilizing intracerebral infection revealed an association between the inflammatory response and blood–labyrinth barrier dysfunction, possibly contributing to hearing loss [365]. The guinea pig model is also directly linked to the inflammatory response and hearing loss [366]. Furthermore, the contribution of macrophage inflammatory protein (MIP-1) to the inflammatory response in the inner ear and hearing loss was established [367]. Nonetheless, the exact mechanisms and possible interventions aimed at preventing hearing loss remain to be determined.

## Understanding immunity to Cmv in tissues: implications for vaccine development

Considering its prevalence in the population and its ability to cause a wide array of morbidities, HCMV is a highly clinically relevant pathogen. Unfortunately, existing antiviral therapies are burdened with significant limitations, including numerous cases of antiviral resistance [3]. The best approach to combat infectious diseases, especially viral diseases, is the use of vaccines; however, despite several decades of intensive research, licensed vaccines for HCMV have not been developed [368]. Different HCMV vaccine candidates, including live attenuated vaccines, adjuvanted protein vaccines that incorporate the viral fusogen gB or gB and the pentameric complex, DNA vaccines coding for gB and a major target of T cells pp65, and mRNA vaccines encoding gB, the pentameric complex, and pp65, as well as different viral vector and peptide vaccines, have been developed thus far and tested or are currently being tested clinically [369]. Although many vaccine candidates remain in various phases of clinical evaluation, numerous candidates have failed to demonstrate success in clinical trials, which is defined as reaching up to 50% efficacy in preventing HCMV acquisition. It is unclear why HCMV vaccine candidates have failed to induce improved immune protection. As persistent viruses, CMVs have coevolved with their hosts and established or adopted numerous immunoregulatory mechanisms that benefit not only the virus [17] but also potentially the host. However, the extent to which viral immunosubversion contributes to the inefficiency of vaccine candidates remains to be determined. Although the remarkable ability of CMV to evade multiple immune mechanisms for viral control and clearance is well known, most of our knowledge about CMV’s immune evasion strategies is restricted to only a few organs, such as the spleen and liver. The extent to which the viral immunosubversion of different arms of the immune system differs among various tissues and cell types remains to be determined. Furthermore, multiple circulating HCMV strains exist and vary enough to allow reinfection of HCMV-seropositive individuals [370, 371]. Thus, a successful vaccine will have to overcome not only immune evasion strategies but also the existence of multiple HCMV strains.

We anticipate that an efficient vaccine will also have to generate immunity that will prevent infection of critical tissues. First, if horizontal transmission is to be reduced, immunity at mucosal sites must be induced at sufficient levels. The example of persistent infection in salivary glands highlights the outstanding adaptability of CMV to the host, enabling prolonged virus shedding and increasing the chances of efficient transfer to new hosts [21]. This feature is pronounced in children, who disseminate the virus for extended periods and are considered to be major HCMV transmitters in the population [372]. The mechanisms of CMV persistence in the salivary glands upon infection in adults have been extensively studied using a mouse model [21], and it has been suggested that targeting protective CD4 T-cell epitopes by vaccination could represent an efficient strategy [263]. However, the underlying mechanisms of prolonged CMV shedding in saliva during early life remain insufficiently understood [244, 373]. Furthermore, although some data exist on why T-cell immunity is inefficient in mucosal tissues in the case of CMV, the understanding of humoral mucosal immunity is somewhat limited. Future studies should not only deepen our understanding of why infected individuals can shed virions over months and years despite the effective generation of antiviral T cells and antibodies but also enable the use of obtained knowledge for the design of better vaccines or vaccination approaches.

Vaccination of the population could reduce the incidence of transplantation-associated HCMV, as it is well established that the incidence is the lowest if there is preexisting immunity to HCMV in organ recipients [3]. However, whether conventional vaccination provides protection in such a scenario is questionable, as intramuscular vaccination generates efficient systemic immunity but less efficient in tissues [374, 375]. Finally, in the case of congenital infection, a reduction in infection rates within the population could potentiate immunity that would prevent virus transmission to the fetus. Congenital infection can occur due to primary infection, infection with another strain (reinfection), or virus reactivation [376]. Thus, vaccination should also enhance immunity at the virus reactivation/infection site and in tissues such as the placenta, where the virus is transferred to the fetus.

Another critical question is whether therapeutic vaccination against CMV could help reduce the latent viral load in HCMV-seropositive individuals, which would also include better control of the reactivating virus. As discussed in this review, the reactivation of a latent CMV is a significant factor contributing to an increase in morbidity burden and mortality rates in immunocompromised patients. Reactivated virus can be transferred to a fetus, and latent infection with HCMV is associated with numerous other conditions, including increased mortality. During aging, an increase in the number of HCMV genomes was observed [377], indicating diminished immune control of HCMV as we age. Thus, vaccinating older individuals could provide beneficial outcomes, similar to vaccination against varicella zoster virus [378]. However, our understanding of CMV latency, reactivation, and immune surveillance of latent infection, including how the virus is controlled in distinct tissues and cell types, is limited. Studies in mice revealed that antibodies can restrain the spread of the virus upon reactivation and that T cells and NK cells can suppress virus reactivation [379, 380]. Thus, efficient stimulation of these components of the immune system by vaccination could improve control of latent infection. Another outstanding question concerns the CMV latency at various cellular sites. Although CD34^+^ progenitor cells are well-recognized sites of HCMV latency, the extent to which HCMV is latent in other cells remains to be determined [15]. Furthermore, the expression of viral genes during latency seems to be generally repressed [15], making targeting latently infected cells difficult.

Put simply, CMV is not just a villain. HCMV boosts immunity in young adulthood, as exemplified by enhanced responses to flu vaccination [381]. Accordingly, it was demonstrated in mice that latent CMV infection provides symbiotic protection from bacterial infections [382]. Finally, CMVs are being developed as vaccine vectors [383]. Thus, harnessing the immunity of tissues through the CMV vaccine could provide additional layers of protection.
